# Evaluation of titanium cranioplasty and polyetheretherketone cranioplasty after decompressive craniectomy for traumatic brain injury: A prospective, multicenter, non-randomized controlled trial

**DOI:** 10.1097/MD.0000000000021251

**Published:** 2020-07-24

**Authors:** Jingguo Yang, Tong Sun, Yikai Yuan, Xuepei Li, Hang Yu, Junwen Guan

**Affiliations:** a Department of Neurosurgery, West China Hospital, Sichuan University; b Health Ministry Key Laboratory of Chronobiology, College of Basic Medicine and Forensic Medicine, Sichuan University; c Institute of Neurology, Sichuan Provincial People's Hospital, University of Electronic Science and Technology of China, Chengdu, Sichuan Province, PR China.

**Keywords:** cranioplasty, polyetheretherketone cranioplasty, prospective study, titanium cranioplasty

## Abstract

**Introduction:**

Cranioplasty following decompressive craniectomy is routinely performed to restore integrity of skull and improve neurological function. However, reconstructing the cranial defect brings many challenges to neurosurgeons and search for ideal implant materials is one of the most controversial issues. Although many studies have compared the outcomes of titanium and polyetheretherketone (PEEK) cranioplasty, yet no prospective study exists to guide the choice of titanium and PEEK materials.

**Methods/design:**

A non-randomized, partially blinded, prospective cohort study is described that comprehensively compares the long-term outcomes of titanium cranioplasty versus PEEK cranioplasty. One hundred forty-five patients for each group will be recruited. Eligible patients are those with cranial defect due to traumatic brain injury (≥ 16 years), defect size is over 25 cm^2^ and they must agree to participate in the trial. Each participant is evaluated before surgery, on discharge, 3, 6, and 12 months after cranioplasty. The primary outcome is the infection, implant failure and implant deformation requiring revision surgery within 12 months. Secondary outcomes include postoperative complication rate, neurological outcomes, motor function, and cosmetic outcome over a 6-month period.

**Discussion:**

Search for ideal implant materials is throughout the history of cranioplasty. This study will provide robust evidence for the choice of cranioplasty materials.

**Trial registration number:**

ChiCTR2000033406

## 1 Introduction

Despite the negative results of DECRA trial and some disappointing message of RESCUEicp,^[1,2]^ decompressive craniectomy (DC) remains the mainstream surgical procedure, dedicated for decreasing intracranial hypertension for neurotrauma patients. Cranioplasty following DC is routinely performed to restore protective barrier and cosmetic appearance while relating to improved neurological function and cognitive outcomes.^[3,4]^ However, cranioplasty can carry high rates of complications, ranging from 15% to 35%.^[5–10]^ Several aspects of cranioplasty could be considered, these aspects include surgical techniques during cranioplasty, time interval between DC and cranioplasty as well as types of materials used for cranial reconstruction.^[11]^

The ideal material used in cranial reconstruction should be biocompatible, resistant to infection, inexpensive and easy to obtain, as well as malleable to fit defects. Several materials have been used to reconstruct cranial defects with different advantages and disadvantages.^[12–14]^ Given the autologous bone fulfills many of the requirements of the ideal reconstruction material, it has always been regarded as gold standard in cranioplasty. However, a unique and common complication after autologous bone cranioplasty is bone flap resorption, and in severe cases it could result in revision surgery and replacement with alloplastic material.^[15,16]^ Therefore, the necessity to search for ideal synthetic materials for cranioplasty was the impetus for this study.

Over time, various materials are considered as an alternative to prevent bone flap resorption and donor site morbidity. Methyl methacrylate was an early used material in cranioplasty, it became useful because of its malleability, lightness, heat resistance, and strength. However, the exothermic reaction during the preparation process may cause burn injuries.^[17]^ Other common materials include hydroxyapatite, alumina ceramics, both have positive and negative characteristics.^[12–14]^

Titanium mesh is one of the most common alloplastic material used in cranioplasty because it has low infection rate, good mechanical strength with low costs. In addition, the titanium mesh prefabricated using 3-dimensional computed tomography could lead to good cosmetic appearance.^[18]^ Nonetheless, titanium mesh also has several disadvantages, it proved that some patients had metal allergies and alternative materials should be used.^[19]^ Also, the erosion of overlying soft tissue and implant exposure is another complication.^[20]^ Finally, titanium mesh is easy to be deformed by external force.

Polyetheretherketone (PEEK) is widely used in current practice, it has the advantages of being biocompatible, chemical inert and radiolucent. In addition, customized patient-specific PEEK implants can be designed using computer-assisted 3D technology and can also be used in complex craniofacial reconstruction.^[21,22]^ Despite these advantages, PEEK implants are expensive and the epidural effusion after cranioplasty trouble many surgeons, and a study speculated that the effusion was because of delayed allergic reactions.^[23]^

Although an increasing number of studies on cranioplasty have been reported, there is a paucity of high-level evidence comparing the outcomes of titanium cranioplasty and PEEK cranioplasty. Therefore, the purpose of this study is to conduct a study to compare long-term outcomes of titanium cranioplasty versus PEEK cranioplasty.

## 2 Methods and design

### 2.1 Objective

The objective of this trial is to compare the long-term outcomes of titanium cranioplasty cohort with that of PEEK cohort in the setting of trauma patients. The primary objective is to compare the rate of implant failure (defined as infection, implant exposure and other causes requiring removal of the implanted material) at any time within 1 year after cranioplasty. The secondary objective is to compare the complication rates and neurological function recover following cranioplasty. Complication events after cranioplasty are investigated within 6 months after surgery and neurological function is evaluated at 3 and 6 months after cranioplasty.

### 2.2 Study design

**Figure 1:**
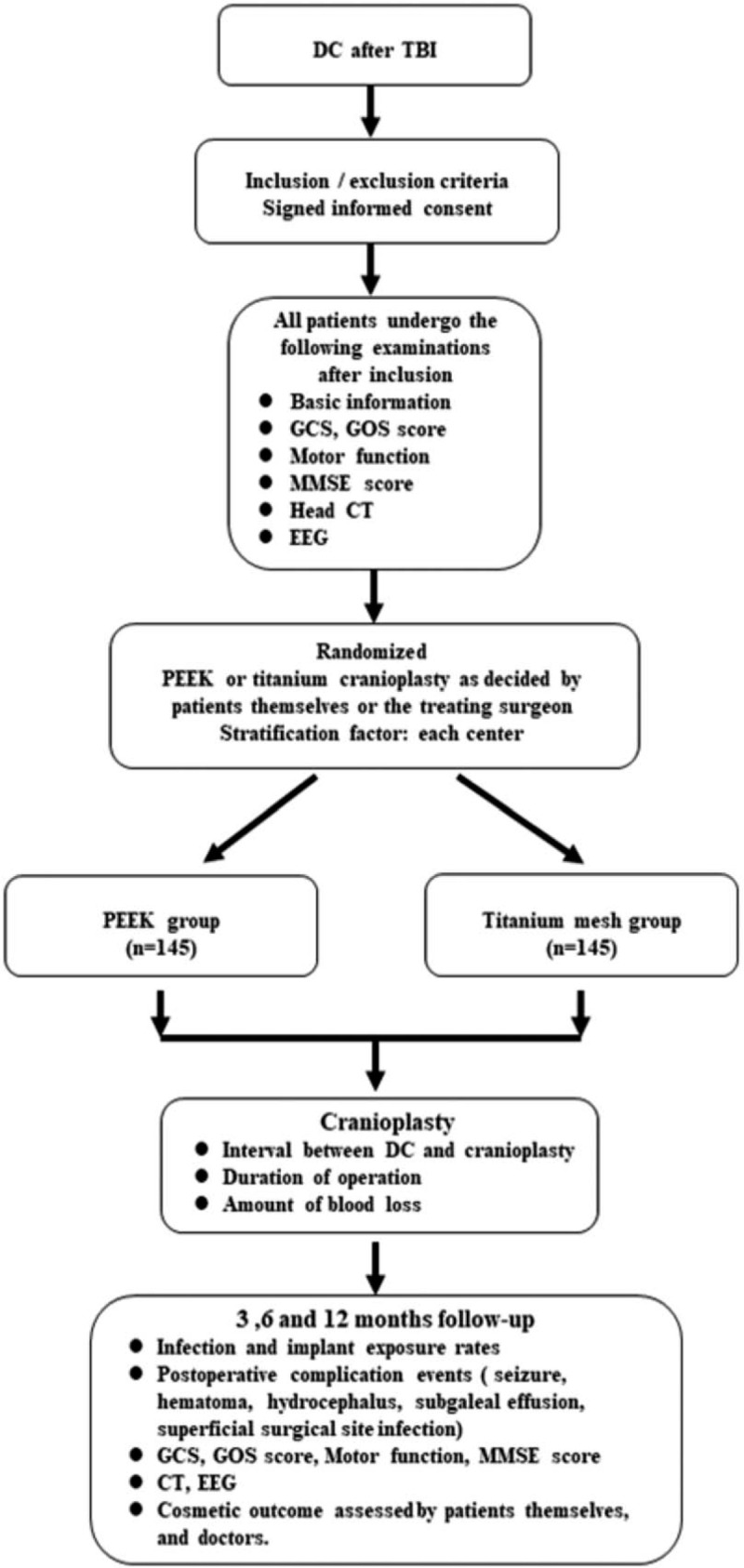
Flow chart of the participants (procedure) through the trial. EEG = electroencephalography, CP = cranioplasty, DC = decompressive craniectomy, GCS = Glasgow Coma Scale, GOS = Glasgow Outcome Scale, MMSE = Mini- Mental State Examination, PEEK = polyetheretherketone, TBI = traumatic brain injury.

The titanium cranioplasty and polyetheretherketone cranioplasty after DC for traumatic brain injury: Phase I (PTCPTBI-1) is a multicenter, partially blinded, non-randomized cohort study involving 20 centers in China. The west China hospital is the leading and coordinating center of this study. One hundred forty-five eligible participants for each group will be recruited. Each participant is evaluated before surgery, 3 months, 6 months and 1 year after surgery by experienced assessors. The patient flowchart is shown in Figure 1.

### 2.3 Participants

Patients are eligible to participate in the PTCPTBI-1 trial if they are diagnosed with cranial defect due to TBI, aged over 16 years, either sex, and the cranial defect size is over 25 cm^2^. Patients must agree to participate in this clinical trial and the informed consent is signed by patients themselves or next of kin on behalf of the patient.

Exclusion criteria are as follows:

1. Patients with DC for other causes (ischemic or hemorrhagic stroke, infiltrative tumor and so on).
2. Prior cranioplasty surgery.
3. Bilateral cranial defect.
4. Hypersensitivity to metals.
5. Suspicion of hydrocephalus.
6. History of radiation therapy.
7. Previous scalp transfer.
8. With intracranial infection or hematoma.
9. Patients with operational contradictions, for example, poor general condition.

### 2.4 Recruitment

Patients are being recruited from 20 hospitals all over China, from July 2020. Two strategies are included in the recruitment process in this trial. First, patients are recruited when the cranioplasty surgery is explained on outpatient department. Second, after reviewing patients’ database, those who receive DC in each hospital will be informed for further visits.

### 2.5 Randomization and blinding

After enrolment and informed consent, patients are assigned to titanium cranioplasty and PEEK cranioplasty groups. The assignment will be decided by treating surgeon or patients themselves. In order to ensure the quality of this trial, blinding is applied to statisticians who will not contact researchers and the assessors involved will also be blinded.

### 2.6 Interventions

#### 2.6.1 Manufacture of titanium and PEEK implants

Patients are scheduled for undergoing imaging procedure (high-resolution computed tomography scan of head) before surgery. Next to the imaging performed, the implanted titanium mesh and PEEK are both designed individually, which could reconstruct bone integrity and achieve bone symmetric. The titanium implant is generated by mold compression with a thickness from 0.6 to 1.0 mm, while the PEEK implant is prefabricated using computer-assisted 3D printing technology. The rage of titanium implant is usually 0.5 to 1 cm larger than the bone defect but the PEEK implant is perfectly matched to the defect size.

#### 2.6.2 Surgical procedure

Neurosurgeons with extensive experience conduct both types of cranioplasties in each center. Since this is a multicenter trial, personnel involved in the study will be trained centrally in advance to achieve unification. The reference surgical techniques were presented in our previous study.^[10]^

#### 2.6.3 Titanium and PEEK cranioplasty

After careful hair shaving before surgery, the patient receives anesthesia. Then, the scalp is vigorously washed and scalp preparation is applied, and care was taken not to damage the scalp and avoid contamination. After preparation, skin and subcutaneous layers are dissected to expose the dura, and during the process dura tearing should be avoided to prevent postoperative cerebrospinal fluid leakage. Secondly, the scalp flap is reflected with scalp hooks, and bleeding is controlled by a bipolar coagulator. Hydrogen peroxide is also used to reduce bleeding and risk of infection. Consideration the titanium should cover the cranial defect, the exposed area is 0.5 to 1.0 cm larger than the skull edge. After debriding the bone margins with a monopolar coagulator, we intensively dissect the temporal muscle using a scissors. The management of temporal muscle is based on neurosurgeons’ experience. The titanium mesh implant is placed under temporal muscle and appropriate adjustment is made to ensure precise position. Next, we suspend the dura and anchor the implant and the wound drain is positioned for drainage of blood above the titanium implant. Finally, the galeal layer and the skin are sutured respectively. The drain is left for about 3 days after surgery and removed appropriately.

### 2.7 Outcomes

As is shown in Table 1, each patient is evaluated before surgery, on discharge, 3, 6, and 12 months after cranioplasty by experienced assessors including neurologists, neurosurgeons.

**Table 1:**
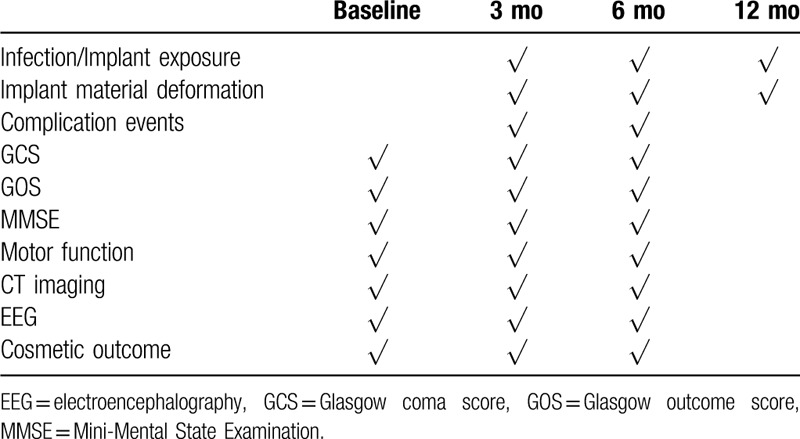
Study schedule.

#### 2.7.1 Primary outcome

The primary outcome is the rate of implant failure 12 months after cranioplasty. The implant failure is referred to the occurrence of infection, implant exposure, deformation of titanium mesh or other causes that require removal of the implanted material.

#### 2.7.2 Secondary outcomes

The secondary outcomes include complications rates, neurological outcomes, motor function, and cosmetic outcomes at the time of discharge, 3 and 6 months after cranioplasty.

1. Complication events occurring in the trial within 6 months include: postoperative new-onset seizure, postoperative intracranial hematoma, postoperative hydrocephalus, subgaleal effusion (the volume of drainage and mechanism will be investigated) and superficial surgical site infection are investigated.
2. Neurological outcomes will be assessed by Glasgow Coma Scale, Glasgow Outcome Scale and Mini-Mental State examination prior to surgery and at discharge, 3 and 6 months after cranioplasty.
3. Motor function is evaluated using Oxford grading system, and 6 grades (0–5) will be assessed before and on discharge, 3 and 6 months during follow-up.
4. Cosmetic outcome after surgery is assessed by patients themselves and neurosurgeons. The degree of cosmetic satisfaction is classified into 4 level: completely satisfactory, satisfactory, fair, unsatisfactory.

### 2.8 Assessment of outcomes

All participants in this trial will be trained centrally, this is done to obtain uniformity when interpreting the results: standardized diagnose of complication events and measurement of neurological outcomes, motor function, and cosmetic appearance. Case report forms are developed to record operative findings and the outcomes.

### 2.9 Sample size

Previously published reports indicated that the complication rate is around 15% for patients with PEEK cranioplasty, compared with a rate of 30% for titanium cranioplasty cohort.^[10]^ We calculate that a sample of 120 for each group will be required in this study with a significance level of 5% (2-sided) and a power of 80% to demonstrate a 15% difference in the rate of complication rates. Considering loss to follow-up, the sample size is enlarged to 290.

### 2.10 Data collection

For all patients, information concerning patient's characteristics (age, gender, indication for DC, comorbidities), basic data and imaging will be recorded. At a later time, experienced staff at each center will collect the baseline information of neurological function, motor function, and cognitive assessment. All patients will stay in follow-up for a minimum of 1 year, and their clinical information will be collected at 3, 6 and 12 months.

When the patients visit the outpatient department, they will be asked to fill out the follow-up questionnaires. If they have difficulties in completing the evaluation, their family members will help them. Data will be anonymized by each center and the data will be transferred to the assessors and transmitted into the electronic database in time. Serious adverse events occurring during the study period are documented.

### 2.11 Data analysis

All data are analyzed using statistical software SPSS version 22 (IBM, Armonk, NY). Probability values (*P*) < .05 is considered to have statistical difference. Categorical variables are described as number (percent). In terms of quantitative data, continuous variables following normal and non-normal distribution are described as arithmetic mean ± standard deviation and median (range), respectively. To compare the 2 groups on implant failure rate and complication rate, Chi-squared test is used (Fisher exact test is used when appropriate). Independent sample *t*-test is used to analyses normal distribution continuous parameters. If the data is non-normal distribution, a non-parameter test, Mann–Whitney *U* test is implemented. Subgroup analysis will be used when appropriate.

### 2.12 Data and safety monitoring

All research procedures are extensively used, common and safe procedures used in clinical cranioplasty practice for cranial defect patient population every day. Therefore, the risk of serious adverse events will be minimal. An independent data monitoring committee will periodically monitor safety of this study and identify any protocol modifications.

### 2.13 Patient and public involvement statement

No patients are involved in the design of this study; however, outcomes will be picked in order to critically and comprehensively examine the efficacy of titanium and PEEK cranioplasty. Participants could contact us if they have emotional needs. A phone call will be sent to all participants after completion of the study. After completion, a journal manuscript will be written to provide feedbacks on the trial results.

## 3 Discussion

Cranioplasty, dating back to 7000 BC, is the most common reconstruction surgery performed with either autologous bone or alloplastic materials after DC for traumatic brain injury. Nowadays, implanted material for cranioplasty is still controversial, which brings challenges to many surgeons. Titanium mesh, a most commonly used alloplastic material, has a high overall strength and malleability. However, a number of studies have demonstrated that use of titanium is along with high rates of complications, such as metal hypersensitivity, erosion of overlying soft tissue and implant exposure.^[19,20]^ In some comparative studies between PEEK and titanium cranioplasty, PEEK is a better implanted option.^[24]^ The data is limited and there is paucity of high-level evidence comparing PEEK and titanium cranioplasty. The PTCPTBI-1 is the first multicenter, non-randomized, partially blinded, prospective cohort study to provide high-level evidence of implant material choice. The primary outcome is implant failure rate within 12 months after surgery and the secondary outcomes include complication rates, neurological functions and cosmetic outcome within 6 months after cranioplasty. The second highlight of this study is to investigate the timing of cranioplasty, which remains controversial in cranioplasty. Finally, subgaleal effusion is a common but less studied complication in previous reports, the underlying mechanism and management will be investigated in this study. Overall, this study will help neurosurgeons choose a better alloplastic material after DC for TBI. One limitation of this study is the varying medical conditions and surgeons’ experiences in multicenter setting; however, personnel involved will get central training to reach uniform standard.

## Author contributions

Jingguo Yang is responsible for providing review of concept, study design, and protocol writing. Junwen Guan conceived the original idea for the study, study design, obtaining funding, edited the paper and is overall guarantors. Tong Sun, Xuepei Li, and Yikai Yuan are involved in trial design and revised the protocol. Hang Yu is responsible for statistical analysis, investigation, and data curation. All authors approve the final manuscript.

## References

[1] Hutchinson PJ Kolias AG Timofeev IS Trial of decompressive craniectomy for traumatic intracranial hypertension. N Engl J Med 2016;375:1119–30. 27602507 10.1056/NEJMoa1605215

[2] Cooper DJ Rosenfeld JV Murray L Decompressive craniectomy in diffuse traumatic brain injury. N Engl J Med 2011;364:1493–502. 21434843 10.1056/NEJMoa1102077

[3] Shahid AH Mohanty M Singla N The effect of cranioplasty following decompressive craniectomy on cerebral blood perfusion, neurological, and cognitive outcome. J Neurosurg 2018;128:229–35. 28298042 10.3171/2016.10.JNS16678

[4] Halani SH Chu JK Malcolm JG Effects of cranioplasty on cerebral blood flow following decompressive craniectomy: a systematic review of the literature. Neurosurgery 2017;81:204–16. 28368505 10.1093/neuros/nyx054

[5] Zanaty M Chalouhi N Starke RM Complications following cranioplasty: incidence and predictors in 348 cases. J Neurosurg 2015;123:182–8. 25768830 10.3171/2014.9.JNS14405

[6] Chaturvedi J Botta R Prabhuraj AR Complications of cranioplasty after decompressive craniectomy for traumatic brain injury. Br J Neurosurg 2016;30:264–8. 26083136 10.3109/02688697.2015.1054356

[7] Gooch MR Gin GE Kenning TJ Complications of cranioplasty following decompressive craniectomy: analysis of 62 cases. Neurosurg Focus 2009;26:E9. 10.3171/2009.3.FOCUS0962 19485722

[8] Chang V Hartzfeld P Langlois M Outcomes of cranial repair after craniectomy. J Neurosurg 2010;112:1120–4. 19612971 10.3171/2009.6.JNS09133

[9] Rosinski CL Patel S Geever B A retrospective comparative analysis of titanium mesh and custom implants for cranioplasty. Neurosurgery 2020;86:E15–22. 31529096 10.1093/neuros/nyz358

[10] Zhang Q Yuan Y Li X A large multicenter retrospective research on embedded cranioplasty and covered cranioplasty. World Neurosurg 2018;112:e645–51. 29374612 10.1016/j.wneu.2018.01.114

[11] Iaccarino C Kolias AG Roumy LG Cranioplasty following decompressive craniectomy. Front Neurol 2020;10:1357. 32063880 10.3389/fneur.2019.01357 PMC7000464

[12] Shah AM Jung H Skirboll S Materials used in cranioplasty: a history and analysis. Neurosurg Focus 2014;36:E19. 24684331 10.3171/2014.2.FOCUS13561

[13] Harris DA Fong AJ Buchanan EP History of synthetic materials in alloplastic cranioplasty. Neurosurg Focus 2014;36:E20. 24684333 10.3171/2014.2.FOCUS13560

[14] Zanotti B Zingaretti N Verlicchi A Cranioplasty: review of materials. J Craniofac Surg 2016;27:2061–72. 28005754 10.1097/SCS.0000000000003025

[15] Malcolm JG Mahmooth Z Rindler RS Autologous cranioplasty is associated with increased reoperation rate: a systematic review and meta-analysis. World Neurosurg 2018;116:60–8. 29753896 10.1016/j.wneu.2018.05.009

[16] van de Vijfeijken SECM Münker TJAG Spijker R Autologous bone is inferior to alloplastic cranioplasties: safety of autograft and allograft materials for cranioplasties, a systematic review. World Neurosurg 2018;117:443–52. 29879511 10.1016/j.wneu.2018.05.193

[17] Spence WT Form-fitting plastic cranioplasty. J Neurosurg 1954;11:219–25. 13163718 10.3171/jns.1954.11.3.0219

[18] Cabraja M Klein M Lehmann TN Long-term results following titanium cranioplasty of large skull defects. Neurosurg Focus 2009;26:E10. 10.3171/2009.3.FOCUS091 19485714

[19] Sun Y Hu Y Yuan Q Association between metal hypersensitivity and implant failure in patients who underwent titanium cranioplasty. J Neurosurg 2018;1:1–7. 10.3171/2018.1.JNS171804 29979123

[20] Yoshioka N Tominaga S Titanium mesh implant exposure due to pressure gradient fluctuation. World Neurosurg 2018;119:e734–9. 30092473 10.1016/j.wneu.2018.07.255

[21] Nguyen PD Khechoyan DY Phillips JH Custom CAD/CAM implants for complex craniofacial reconstruction in children: our experience based on 136 cases. J Plast Reconstr Aesthet Surg 2018;71:1609–17. 30220563 10.1016/j.bjps.2018.07.016

[22] Hanasono MM Goel N DeMonte F Calvarial reconstruction with polyetheretherketone implants. Ann Plast Surg 2009;62:653–5. 19461279 10.1097/SAP.0b013e318184abc7

[23] Qiu S You W Wang H Allergic epidural effusion following polyetheretherketone cranioplasty. J Craniofac Surg 2019;30:e241–3. 30730518 10.1097/SCS.0000000000005192

[24] Zhang J Tian W Chen J The application of polyetheretherketone (PEEK) implants in cranioplasty. Brain Res Bull 2019;153:143–9. 31425730 10.1016/j.brainresbull.2019.08.010

